# The Fatty Liver Index: a simple and accurate predictor of hepatic steatosis in the general population

**DOI:** 10.1186/1471-230X-6-33

**Published:** 2006-11-02

**Authors:** Giorgio Bedogni, Stefano Bellentani, Lucia Miglioli, Flora Masutti, Marilena Passalacqua, Anna Castiglione, Claudio Tiribelli

**Affiliations:** 1 Centro Studi Fegato (Liver Research Center), AREA Science Park, Basovizza, Trieste, and Department of Biochemistry, Biophysics and Macromolecular Chemistry, University of Trieste, Trieste, Italy; 2 Nutrition and Liver Center, AUSL Modena, Carpi Hospital, Carpi, Modena, Italy

## Abstract

**Background:**

Fatty liver (FL) is the most frequent liver disease in Western countries. We used data from the Dionysos Nutrition & Liver Study to develop a simple algorithm for the prediction of FL in the general population.

**Methods:**

216 subjects with and 280 without suspected liver disease were studied. FL was diagnosed by ultrasonography and alcohol intake was assessed using a 7-day diary. Bootstrapped stepwise logistic regression was used to identify potential predictors of FL among 13 variables of interest [gender, age, ethanol intake, alanine transaminase, aspartate transaminase, gamma-glutamyl-transferase (GGT), body mass index (BMI), waist circumference, sum of 4 skinfolds, glucose, insulin, triglycerides, and cholesterol]. Potential predictors were entered into stepwise logistic regression models with the aim of obtaining the most simple and accurate algorithm for the prediction of FL.

**Results:**

An algorithm based on BMI, waist circumference, triglycerides and GGT had an accuracy of 0.84 (95%CI 0.81–0.87) in detecting FL. We used this algorithm to develop the "fatty liver index" (FLI), which varies between 0 and 100. A FLI < 30 (negative likelihood ratio = 0.2) rules out and a FLI ≥ 60 (positive likelihood ratio = 4.3) rules in fatty liver.

**Conclusion:**

FLI is simple to obtain and may help physicians select subjects for liver ultrasonography and intensified lifestyle counseling, and researchers to select patients for epidemiologic studies. Validation of FLI in external populations is needed before it can be employed for these purposes.

## Background

Fatty liver (FL) is the most frequent liver disease in Western countries [1-4]. Obesity and its complications, especially type 2 diabetes and hypertriglyceridemia, are likely to be the main responsible of the current epidemic of FL, while ethanol intake may play a minor role [5-7]. In a nested case-control study of the Dionysos Project, we found that body mass index (BMI) was a stronger risk factor for FL than ethanol intake in the general population of Northern Italy [6]. Interestingly, this finding was confirmed by a recent study performed in China [8]. Waist circumference has long been hypothesized to be a predictor of FL independently from BMI, but data for the general population were not available until very recently [1,8]. Because BMI is a surrogate index of body adiposity [9], direct indexes of adiposity such as skinfolds can be of value when studying the relationship between body fatness *per se *and disease [10,11]. Hyperinsulinemia and insulin resistance are common in subjects with FL independently from BMI and thus are expected to be markers of FL in the general population [12]. Despite the operational separation of FL into alcoholic and non-alcoholic fatty liver disease (NAFLD) [4], the relative contribution of ethanol intake and other factors in the pathogenesis of FL is still uncertain [3]. Using data collected during the Dionysos Nutrition & Liver Study [1], we evaluated the contribution of ethanol intake, anthropometry, liver enzymes and metabolic parameters to the risk of FL and developed an algorithm for the prediction of FL in the general population.

## Methods

### Study design

The protocol of the Dionysos Nutrition & Liver Study was described in detail elsewhere [1]. Briefly, of 5780 residents of Campogalliano (Modena, Italy) aged 18 to 75 years, 3345 (58%) agreed to participate to the study; 3329 (99%) of them had all the data required by the Dionysos Project [7,13] and were considered for further analysis. 497 (15%) of them had suspected liver disease (SLD) according to at least one of the following criteria: 1) alanine transaminase (ALT) > 30 U*L^-1^; 2) gamma-glutamyl-transferase (GGT) > 35 U*L^-1^; 3) presence of hepatitis B surface antigen (HBsAg); 4) presence of Hepatitis C (HCV) virus ribonucleic acid (RNA) after detection of anti-HCV antibodies. The 497 subjects with SLD were matched with an equal number of subjects of the same age and sex but without SLD, randomly selected among the remaining 2832 subjects. After exclusion of subjects with HBV or HCV infection, the original analysis was performed on 224 subjects with and 287 without SLD [1]. The present analysis is performed on 216 (96%) subjects with and 280 (97%) without SLD, based on the availability of skinfold measurements.

### Methods

Besides a clinical and laboratory evaluation, each subject underwent a liver ultrasonography, an anthropometric assessment and a 7-day diary of food intake (7DD) [1]. HBsAg and anti-HCV antibodies were assessed and subjects with anti-HCV antibodies underwent an HCV-RNA assessment to confirm HCV infection [1,14]. ALT, aspartate transaminase (AST), GGT, glucose, triglycerides and cholesterol were measured by standard laboratory methods after 8-hr fasting. Insulin was measured by radio-immuno-assay (ADVIA Insulin Ready Pack 100, Bayer Diagnostics, Milan, Italy), with intra- and inter-assay coefficients of variation < 5%. FL was diagnosed by the same operator at ultrasonography [6]. Weight, stature, circumferences (waist and hip) and skinfolds (triceps, biceps, subscapular and suprailiac) were measured by two trained dietitians who had been standardized before and during the study according to standard procedures [15]. Body mass index (BMI) was calculated as weight (kg)/stature (m)^2 ^and the sum of 4 skinfolds by summing triceps, biceps, subscapular and suprailiac skinfolds [16,17]. The 7DD was administered to the subjects by two trained dietitians, who discussed it with the subject when she/he returned it one week later [18]. To avoid the confounding effect of seasonality on food intake, the 7DD diary was administered to a similar number of patients with and without SLD each month [19]. Mean daily ethanol intake was calculated as the mean value of ethanol intake as assessed by the 7DD [20]. The study protocol was approved and supervised by the Scientific Committee of the Fondo per lo Studio delle Malattie del Fegato (Trieste, Italy), and all subjects gave their written informed consent to participate.

### Statistical analysis

Continuous variables are given as medians and interquartile ranges (IQR) because of skewed distributions. Comparisons of continuous variables between subjects with and without FL were performed with the Mann-Whitney test and those of nominal variables with the Fisher's exact test. To identify candidate predictors of FL, we performed a stepwise logistic regression analysis on 1000 bootstrap samples of 496 subjects (probability to enter = 0.05 and probability to remove = 0.1) [21]. All variables besides gender were evaluated as continuous predictors. Linearity of logits was ascertained using the Box-Tidwell procedure [22]. To obtain a linear logit, we transformed age using the coefficient suggested by the Box-Tidwell procedure [(age/10) ^4.9255^] and ALT, AST, GGT, insulin and triglycerides using natural logarithms (log_e_). The logits of the other predictors (BMI, waist circumference, glucose, cholesterol, ethanol and the sum of 4 skinfolds) were linear.

Candidate predictors identified at bootstrap analysis were evaluated using three stepwise logistic models before obtaining a final prediction model (probability to enter = 0.01 and probability to remove = 0.02; these more stringent levels were used to protect against type I errors). The goodness of fit of the models was evaluated using the Hosmer-Lemeshow statistic and their accuracy was assessed by calculating the non-parametric area (AUC) under the receiver-operating curve (ROC) with 95% confidence intervals (95%CI) [23,24]. The standard errors of the regression coefficients of the final model were calculated using 1000 bootstrap samples of 496 subjects. The probabilities obtained from the final model were multiplied by 100 to obtain the fatty liver index (FLI). The sensitivity (SN), specificity (SP), positive likelihood ratio (LR+) and negative likelihood ratio (LR-) of 10-value intervals of FLI were calculated [23]. Statistical analysis was performed using STATA 9.2 (StataCorp, College Station, Texas, USA).

## Results

Table 1 gives the characteristics of the subjects with and without FL. FL was more frequent among males than females (54 *vs. *34%). Age, ethanol intake and cholesterol did not differ between subjects with and without FL. On the contrary, ALT, AST, GGT, BMI, waist circumference, the sum of 4 skinfolds, glucose, insulin and triglycerides were significantly higher in subjects with than in those without FL.

**Figure 1:**
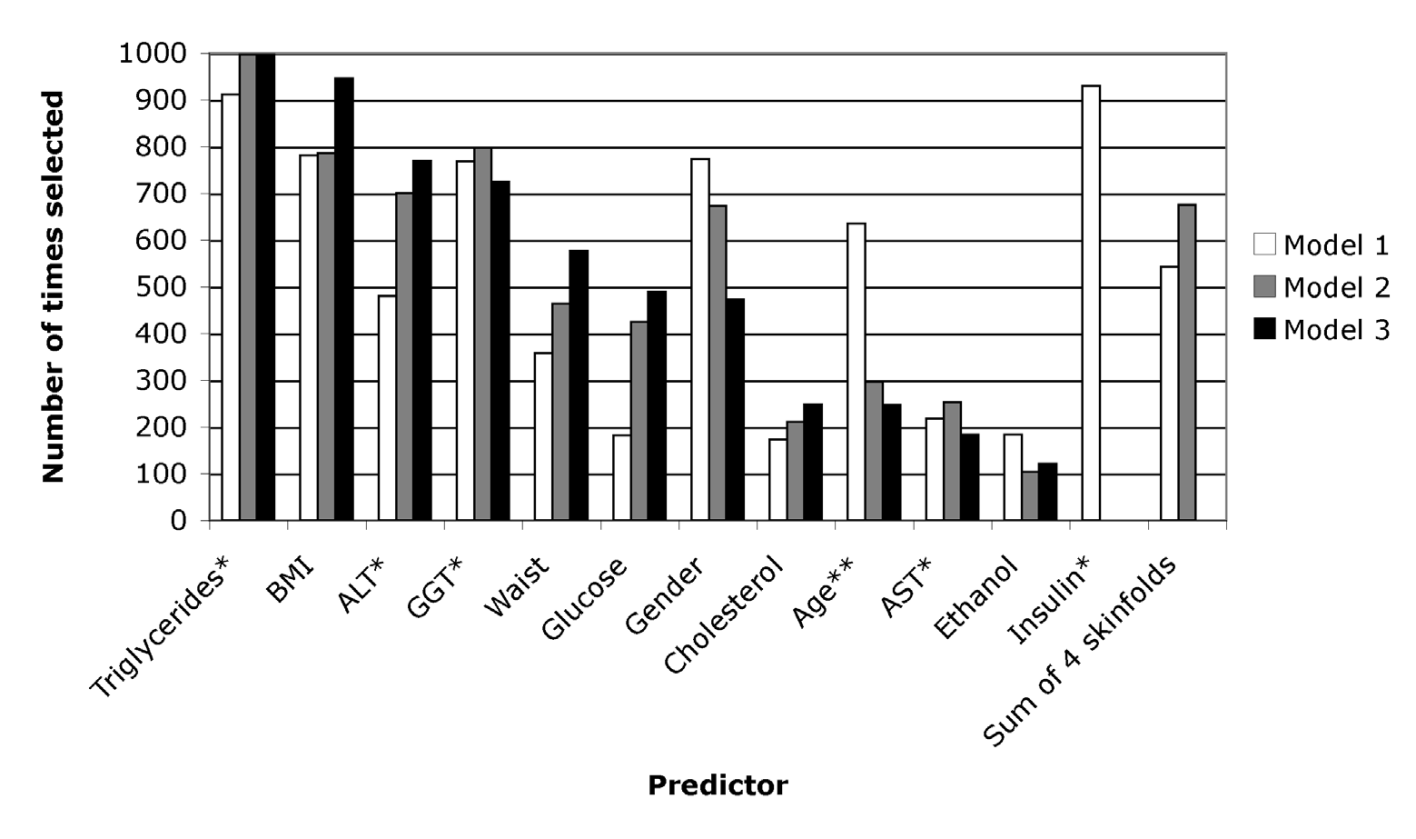
Selection of candidate predictors at bootstrapped stepwise logistic regression. Bars indicate the number of times out of 1000 that the variables were selected for inclusion in 3 models. Model 1 is the starting model, Model 2 removes insulin and Model 3 removes skinfolds. Data are sorted using Model 3. Abbreviations: * = transformed using natural logarithm; ** = transformed using Box-Tidwell transformation (see text for details); other abbreviations as in Table 1.

Figure 1 (Model 1) gives the number of times each of the 13 variables of interest was selected by bootstrapped stepwise logistic regression. The predictors identified most frequently were insulin (93%), triglycerides (91%), BMI (78%), gender (77%), GGT (77%) and age (64%). When these 6 predictors were entered into the stepwise model, age did not remain in the model (*p *= 0.0766; model not shown). The model based on the remaining 5 predictors fitted well (*p *= 0.9976, Hosmer-Lemeshow statistic) and had a ROC-AUC of 0.85 (95%CI 0.82–0.89; model not shown).

Since insulin is not routinely measured, we tested whether its removal from the model would decrease the accuracy of the estimate. After exclusion of insulin, the predictors most frequently identified were triglycerides (100%), GGT (80%), BMI (79%), ALT (70%), the sum of 4 skinfolds (68%) and gender (67%) (Figure 1, Model 2). When these 6 predictors were entered into the stepwise model, ALT did not enter it (*p *= 0.0780; model not shown). The model based on the 5 remaining predictors fitted well (*p *= 0.9704, Hosmer-Lemeshow statistic) and had a ROC-AUC of 0.85 (95%CI 0.81–0.88; model not shown).

Since skinfolds are not routinely measured, we tested whether their removal from the model would decrease the accuracy of the estimate. After exclusion of the sum of 4 skinfolds, the predictors identified most frequently were triglycerides (100%), BMI (95%), ALT (77%), GGT (73%) and waist circumference (58%) (Figure 1, Model 3). When these 5 predictors were entered into the stepwise model, ALT did not enter it (*p *= 0.0241; *p *to remove = 0.0200; model not shown). The model based on the remaining 4 predictors fitted well (*p *= 0.9704, Hosmer-Lemeshow statistic) and had a ROC-AUC of 0.85 (95%CI 0.81–0.88; model not shown).

A comparison of the ROC-AUCs of Models 2 (*p *= 0.6320; Bonferroni's correction) and 3 (*p *= 0.1038) *vs. *Model 1 revealed no difference so that we choose Model 3 for further analysis. The bootstrapped regression coefficients of Model 3 are given in Table 2. We multiplied the probabilities generated by Model 3 per 100 to obtain a score comprised between 0 and 100, which we call the "fatty liver index" (FLI). FLI is thus calculated as:

FLI = (e ^0.953*loge (triglycerides) + 0.139*BMI + 0.718*loge (ggt) + 0.053*waist circumference - 15.745^) /   (1 + e ^0.953*loge (triglycerides) + 0.139*BMI + 0.718*loge (ggt) + 0.053*waist circumference - 15.745^) * 100  

As shown by the standardized regression coefficients, the greatest contribution to the prediction of FL came from waist circumference, followed by BMI, triglycerides and GGT. Table 3 gives the SN, SP, LR+ and LR- for 10-unit intervals of FLI. A FLI < 30 can be used to rule out (SN = 87%; LR- = 0.2) and a FLI ≥ 60 to rule in hepatic steatosis (SP = 86%; LR+ = 4.3).

## Discussion

We used data from the Dionysos Nutrition & Liver Study to develop a simple algorithm for the prediction of FL. Age was not associated with FL in any of the multivariable models while gender lost its association with FL after exclusion of insulin and skinfolds. Ethanol intake was not associated with FL in any of the models. Thus, at least at the values of intake observed in the Dionysos Nutrition & Liver Study, ethanol is not a risk factor for FL in the general population of Northern Italy.

Waist circumference and BMI were the strongest predictors of FL in the final model. Together with the lack of association of FL with ethanol intake, this finding strongly supports the hypothesis that obesity is the main responsible of the current epidemic of FL [1,4,6]. It is of some interest that waist circumference did not add to the prediction of FL when skinfolds were in the model but, from a practical viewpoint, there is no need to measure skinfolds for predicting FL.

Among liver enzymes, only GGT was an independent predictor of FL while AST was not associated with FL in any of the models and ALT was not an independent predictor of FL. We have previously shown that ALT is not a surrogate marker of NAFLD and the present study extends this consideration to the entire spectrum of FL disease [1].

Insulin was the predictor most frequently selected for inclusion in Model 1 and was the second most important predictor after BMI in the same model (data not shown). Thus, we confirm that insulin is an independent risk factor for FL in the general population [12]. It is of some interest that waist circumference did not add to the prediction of FL when insulin was in the model but that it was the strongest predictor of FL in the final model. This cannot be easily explained by the known association between waist and insulin (resistance) because BMI is similarly correlated with this latter [25,26] as also observed in this study (data not shown). Triglycerides were independent predictors of FL in all models, confirming our previous findings [16]. Glucose and cholesterol were not predictors of FL even if it may be noticed that the selection of glucose as potential predictor increased after exclusion of insulin from the model.

The main limitations of the Dionysos Nutrition & Liver Study are the suboptimal respondent rate (58%) and the fact that ultrasonography cannot detect steatohepatitis (SH) [1]. This latter diagnosis can be obtained only by biopsy and, because of obvious ethical reasons, a SH score will never be available in a representative sample of the general population [3]. Scores developed in clinical series may be used for this purpose but they have not been tested in the general population [27,28].

## Conclusion

The "fatty Liver index" (FLI) we developed is accurate and easy to employ as BMI, waist circumference, triglycerides and GGT are routine measurements in clinical practice [7,29,30]. In our population, a FLI < 30 ruled out and a FLI ≥ 60 ruled in hepatic steatosis as detected by ultrasonography. Potential clinical uses of FLI include the selection of subjects to be referred for ultrasonography and the identification of patients for intensified lifestyle counseling [30,31]. On the research side, FLI may be used to select subjects at greater risk of FL for planning observational or interventional studies [30,32]. Even though, for reasons of biological plausibility and coherence with previous studies [5,6,8], we expect that the parameters employed by FLI will be predictors of FL in Western countries besides Italy, it is very important that FLI be validated in external populations before it is employed in practice.

## Abbreviations

7DD = 7-day diary

95%CI = 95% confidence intervals

ALT = Alanine transaminase

Anti-HCV = Antibodies against hepatitis C virus

AST = Aspartate transaminase

AUC = Area under the curve

BMI = Body mass index

FL = Fatty liver

FLI = Fatty liver index

GGT = Gamma-glutamyl-transferase

HbsAg = Hepatitis B surface antigen

HBV = Hepatitis B virus

HCV = Hepatitis C virus

HCV-RNA = Hepatitis C ribonucleic acid

IQR = Interquartile range

LR+ = Positive likelihood ratio

LR- = Negative likelihood ratio

NAFLD = Non-alcoholic fatty liver disease

ROC = Receiver-operating curve

SH = Steatohepatitis

SN = Sensitivity

SP = Specificity

SLD = Suspected liver disease

## Competing interests

The authors declare that they have no competing interests.

## Authors' contributions

GB co-designed the Dionysos Nutrition & Liver Study, analyzed the data and wrote the manuscript; SB and CT co-designed the Dionysos Nutrition & Liver Study and contributed to the manuscript; LM, FM and AC performed the medical evaluation of subjects; MP performed the nutritional assessment of subjects. All authors read and approved the manuscript.

## Pre-publication history

The pre-publication history for this paper can be accessed here:



## Figures and Tables

**Table 1:** Measurements of subjects with and without fatty liver.

	FL (*n *= 228)	No FL (*n *= 268)	*p*
Gender (male/female, *n*)	164/64	141/127	<0.0001
Age (years)	58 (17)	57 (26)	0.9535
Ethanol (g*day^-1^)	13 (35)	11 (28)	0.1272
ALT (U*L^-1^)	27 (21)	19 (16)	<0.0001
AST (U*L^-1^)	22 (10)	20 (8)	0.0004
GGT (U*L^-1^)	31 (35)	19 (14)	<0.0001
BMI (kg*m^-1^)	29.5 (5.8)	25.7 (4.1)	<0.0001
Waist circumference (cm)	98 (16)	86 (14)	<0.0001
Sum of 4 skinfolds (mm)	74.1 (37.4)	59.6 (26.8)	<0.0001
Glucose (mg*dL^-1^)	96 (18)	89 (13)	<0.0001
Insulin (mU*L^-1^)	9 (8)	5 (4)	<0.0001
Triglycerides (mg*dL^-1^)	141 (102)	91 (60)	<0.0001
Cholesterol (mg*dL^-1^)	219 (57)	212 (52)	0.1547

Values are medians and interquartile ranges for continous variables and number of subjects for categorical variables.

Abbreviations: FL = fatty liver; *p *= p-value (Mann-Whitney *U*-test for continuous variables and Fisher's Exact test for categorical variables); ALT = alanine transaminase; AST = aspartate transaminase; GGT = gamma-glutamyl-transferase; BMI = body mass index.

**Table 2:** The parameters of the fatty liver index (FLI).

	β	SE (β)	STD (β)	*p*
Log_e _(triglycerides, mg*dL^-1^)	0.953	0.211	0.308	<0.0001
BMI (kg*m^2-1^)	0.139	0.050	0.353	0.006
Log_e _(GGT, U*L^-1^)	0.718	0.202	0.278	<0.0001
Waist circumference (cm)	0.053	0.019	0.356	0.005
Constant	-15.745	1.631	-	<0.0001

Abbreviations: β = regression coefficient; SE = standard error; STD = standardized value; log_e _= nathural logarithm. Other abbreviations as in Table 1. FLI is calculated by multiplying the predicted probabilities per 100 (see text for the formula).

**Table 3:** Diagnostic accuracy of the fatty liver index.

FLI cut-point	%	SN	SP	LR+	LR-
≥10	90	98	17	1.2	0.1
≥20	74	94	44	1.7	0.1
**≥30**	**60**	**87**	**64**	**2.4**	**0.2**
≥40	53	82	72	2.9	0.3
≥50	43	70	80	3.5	0.4
**≥60**	**36**	**61**	**86**	**4.3**	**0.5**
≥70	28	49	91	5.2	0.6
≥80	18	35	96	9.3	0.7
≥90	9	18	99	15.6	0.8

Abbreviations: FLI = fatty liver index; % = number of patients with FLI ≥ cut-point; SN = sensitivity; SP = specificity; LR+ = positive likelihood ratio; LR- = negative likelihood ratio.
